# Supplementary material for: Rapid proliferation due to better metabolic adaptation results in full virulence of a filament-deficient Candida albicans strain
Source: Nat Commun. 2021 Jun 23;12:3899. doi: 10.1038/s41467-021-24095-8 (PMC8222383; doi:10.1038/s41467-021-24095-8)
Supplement: Supplementary file 1 — Supplementary Information [file 41467_2021_24095_MOESM1_ESM.pdf]

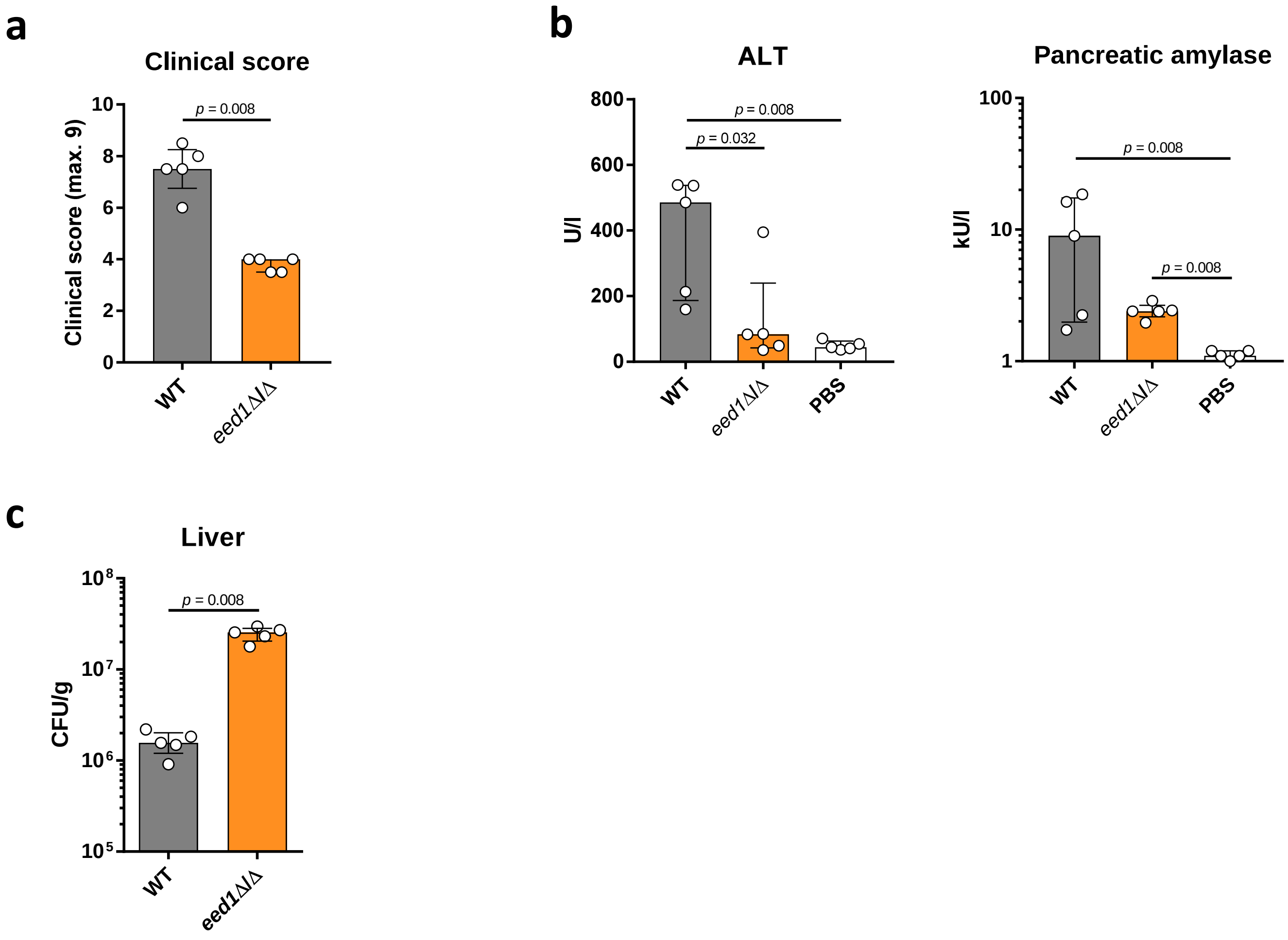

Supplementary Fig. 1 An *eed1*Δ/Δ mutant is attenuated in damage and virulence potential in the intraperitoneal infection model 24 h post infection. Mice were infected intraperitoneally with $1x10^8$ cells of the WT (SC5314), or *eed1*Δ/Δ mutant or were mock infected with PBS (n=5 per group). a) The semiquantitative clinical score was determined by assessing fur, coat and posture, behavior and lethargy, fibrin exudation and other symptoms like diarrhea. The score ranges from 0 (no symptoms) to 10 (severe illness). b) Damage of liver and pancreas was quantified by measuring serum levels of alanine aminotransaminase (ALT) and pancreatic amylase, respectively. c) Fungal burden of the liver. a-c) Data are shown as median with interquartile range and were statistically analyzed using the two-sided Mann-Whitney test, *p*-values are shown in the graphs. Source data are provided as a Source Data file.

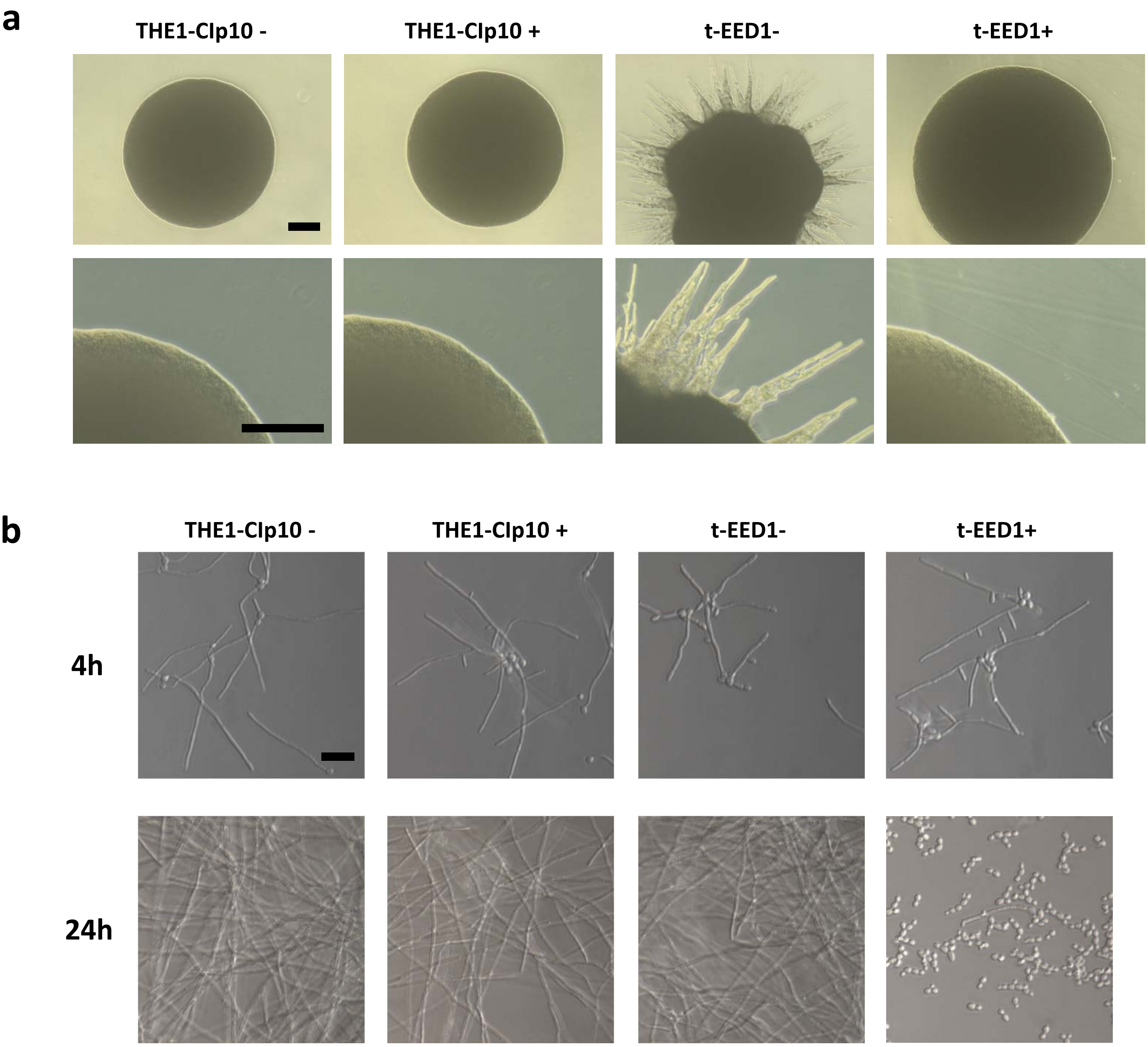

Supplementary Fig. 2 In the absence of doxycycline t-EED1 is hyper-filamentous on solid medium but forms filaments comparable to the WT in liquid medium. Representative pictures of three biologically independent experiments are shown. a) Colony morphology of *C. albicans* WT (THE1-CIp10) and t-EED1 grown for 2 days on YPD agar at 25 °C in the presence (+) or absence (-) of doxycycline. Bars represent 200 µm (upper row) and 100 µm (lower row). b) Morphology of *C. albicans* WT (THE1-CIp10) and t-EED1 grown for 4 h and 24 h in RPMI at 37 °C and 5% CO_2 in the presence (+) or absence (-) of doxycycline. Scale bar represents 20 µm and applies to all images.

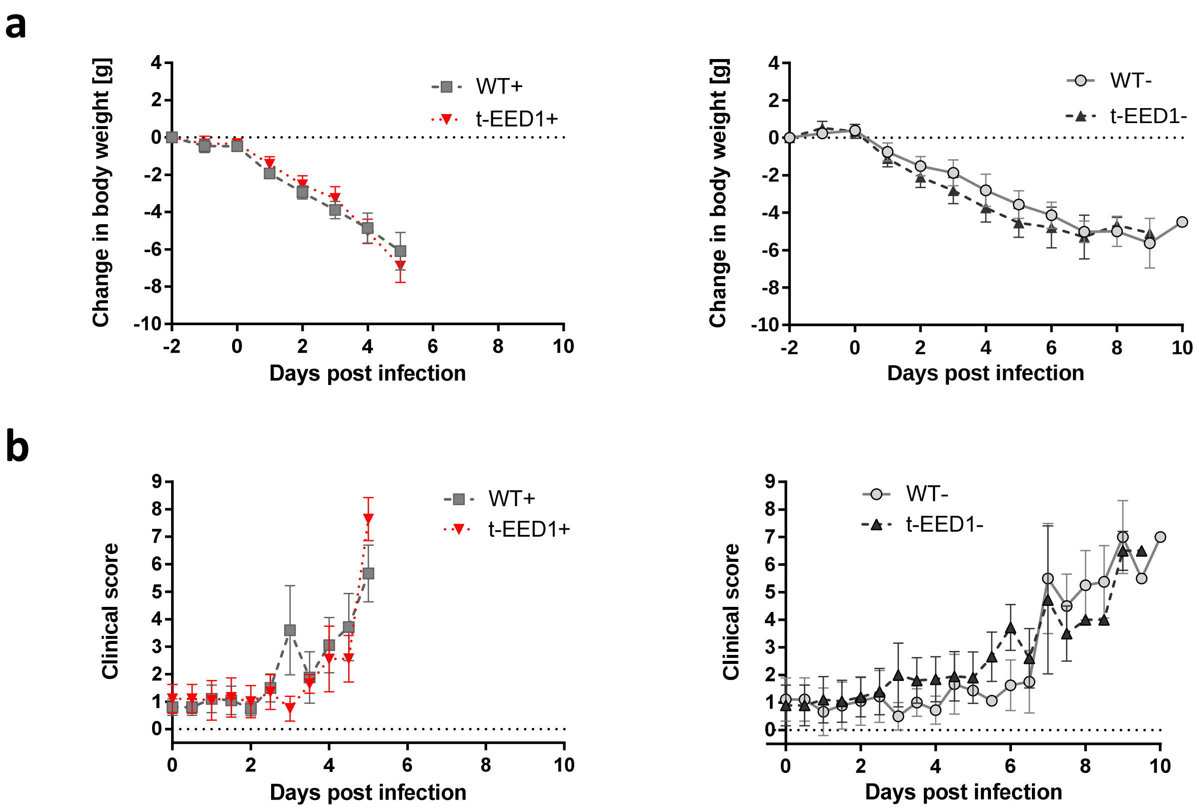

Supplementary Fig. 3 Development of clinical symptoms and change in body weight during the course of systemic infection with WT and t-EED1. Mice were intravenously infected with 2.5x10^4 CFU/g body weight with WT (THE1-Clp10) or t-EED1 in the presence (+) or absence of doxycycline (-) supplied to mice via the drinking water. a) Change in body weight relative to body weight at day-3 (start of doxycycline treatment). At day 0, mice were infected intravenously with the indicated *C. albicans* strains. b) Development of clinical symptoms during the course of infection. The semiquantitative clinical score was determined by assessing fur, body surface temperature, behavior and lethargy. The score ranges from 0 (no symptoms) to 8 (severe illness). a,b) Shown is the mean ± SD. One experiment, n=10 mice per group. The surviving mouse in the WT- group (Fig. 3c) was excluded from analysis.

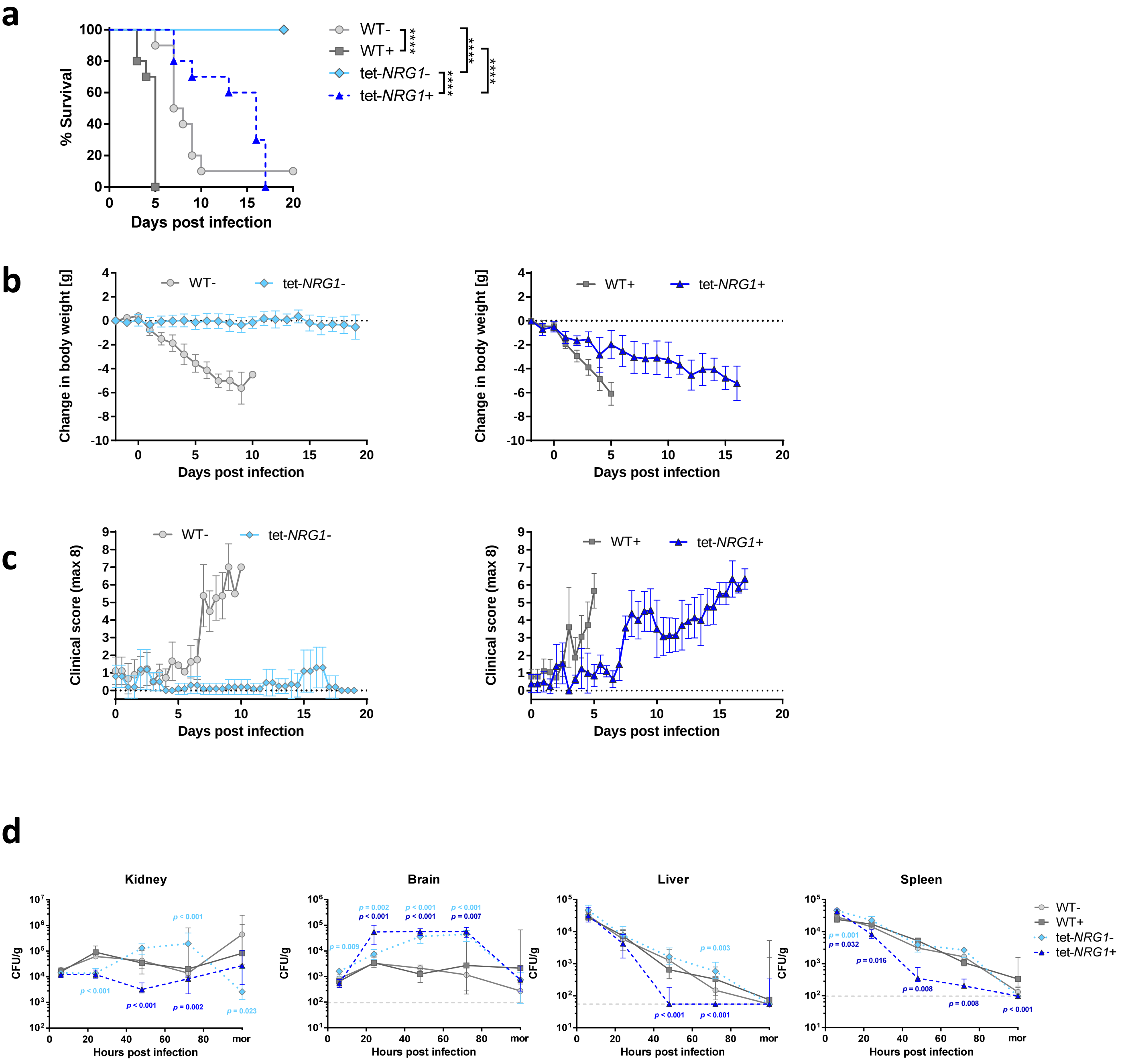

Supplementary Fig. 4: tet-*NRG1*- yeast are avirulent whereas tet-*NRG1*+ hyphae lead to delayed mortality in a mouse model of systemic candidiasis. Mice were intravenously infected with 2.5×10^4 CFU/g body weight with WT (THE1-Clp10) or tet-*NRG1* in the presence (+) or absence (-) of doxycycline supplied to mice via the drinking water. a) Survival was monitored over a period of 20 days and is shown as Kaplan-Meyer curve. Survival curves were analyzed using the two-sided Log-rank (Mantel-Cox) test, two curves were compared at a time; **** $p < 0.0001$. b) Change in body weight relative to body weight at day -3 (start of doxycycline treatment). At day 0, mice were infected intravenously with the indicated *C. albicans* strains. c) Development of clinical symptoms during the course of infection. The semiquantitative clinical score was determined by assessing fur, body surface temperature, behavior and lethargy. The score ranges from 0 (no symptoms) to 8 (severe illness). a-c) One experiment, n=10 mice per group. b,c) Shown is the mean ±SD. The surviving mouse in the WT- group was excluded from analysis. d) Organ fungal loads in mice sacrificed 6, 24, 48 and 72 h post infection and in moribund mice (mor). Mice challenged with tet-*NRG1*- were humanely sacrificed 19 days post infection. Data are shown as median with interquartile range. Dashed lines indicate limit of detection. Moribund: one experiment, n=10; kidney: two independent experiments, n=10; liver and brain: two independent experiments, n=10; spleen: one experiment, n=5. Two-sided Mann-Whitney test, significant differences at time points are indicated by asterisks: light blue, tet-*NRG1*- yeast compared to WT-; dark blue, tet-*NRG1*+ filamentous cells compared to WT+. Source data are provided as a Source Data file.

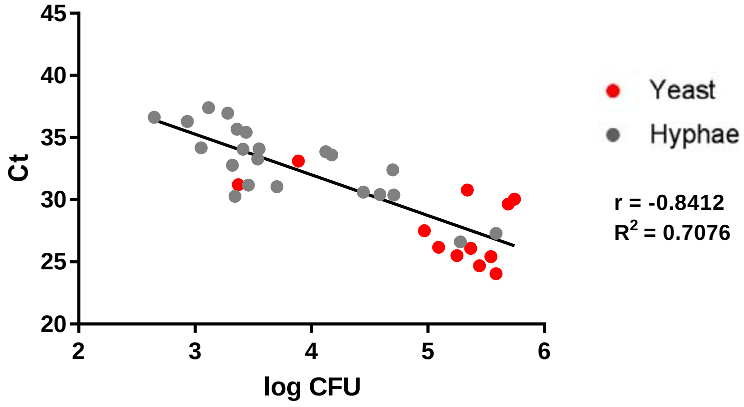

Supplementary Fig. 5 Correlation between fungal DNA content and CFU count from homogenized renal tissues of intravenously infected mice. Renal tissue was homogenized for enumeration of CFU and for DNA isolation to quantitative fungal DNA by PCR, targeting the *C. albicans* 18S rRNA gene *RDN18*. The resulting cycle threshold (Ct) values were plotted against the log transformed CFU counts from the respective samples. A strong correlation (R^2=0.7076) between CFU count and DNA content irrespective of the fungal morphology was observed. Source data are provided as a Source Data file.

Kidney infiltrating leukocytes 24 h p.i.

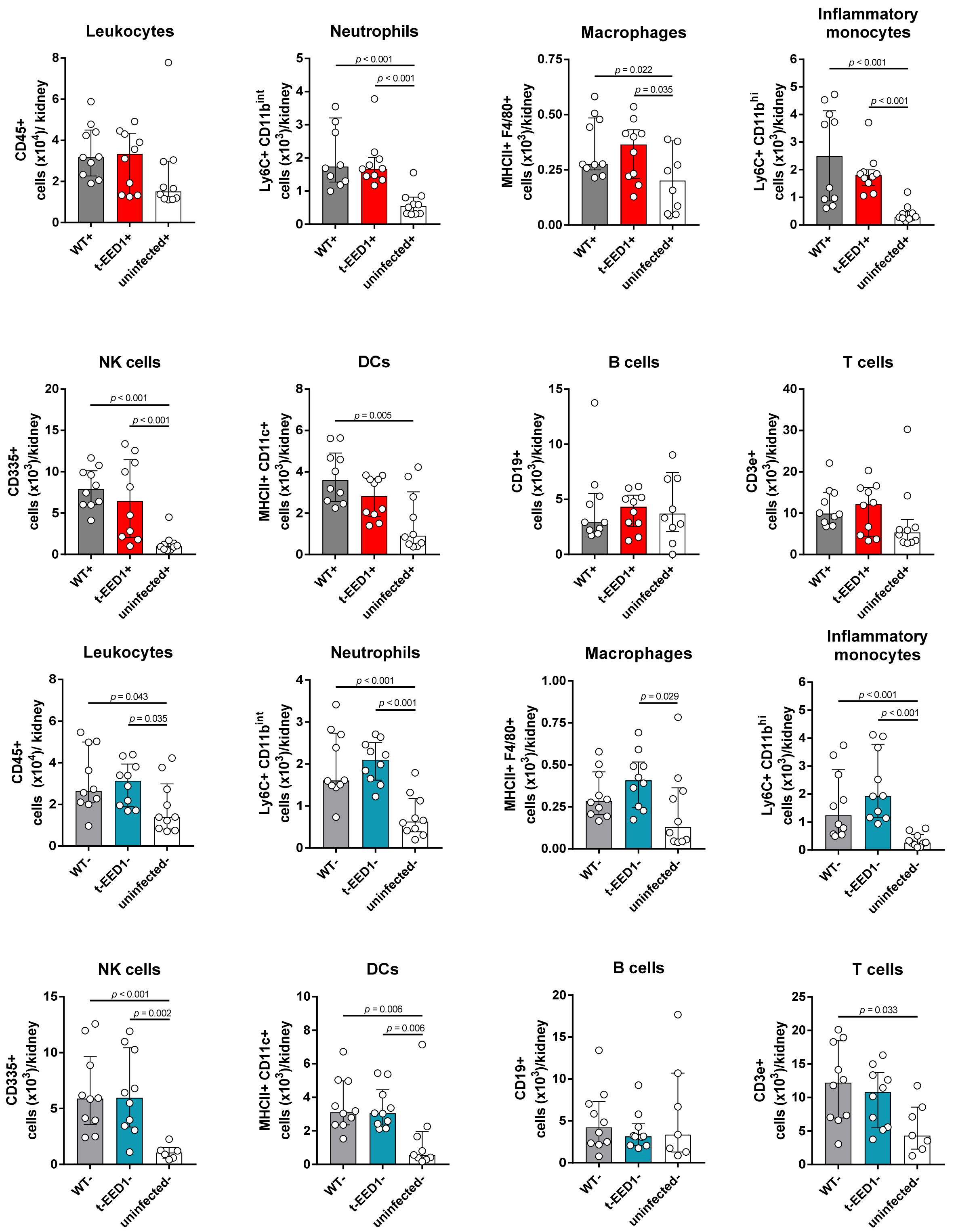

Supplementary Fig. 6 Renal leukocyte infiltration in mice intravenously infected with 2.5x10^4 CFU/g body weight of WT or t-EED1 and in uninfected controls in the presence (+) or absence of doxycycline (-) 24 h post infection. The absolute number of living immune cells per kidney is shown as median and interquartile range. Two independent experiments , n=10, except for uninfected- NK cells n=6, DCs n=9, B cells and T cells n=7. Two-sided Mann-Whitney test, *p*-values are shown in the graphs. Source data are provided as a Source Data file.

Kidney infiltrating leukocytes 48 h p.i.

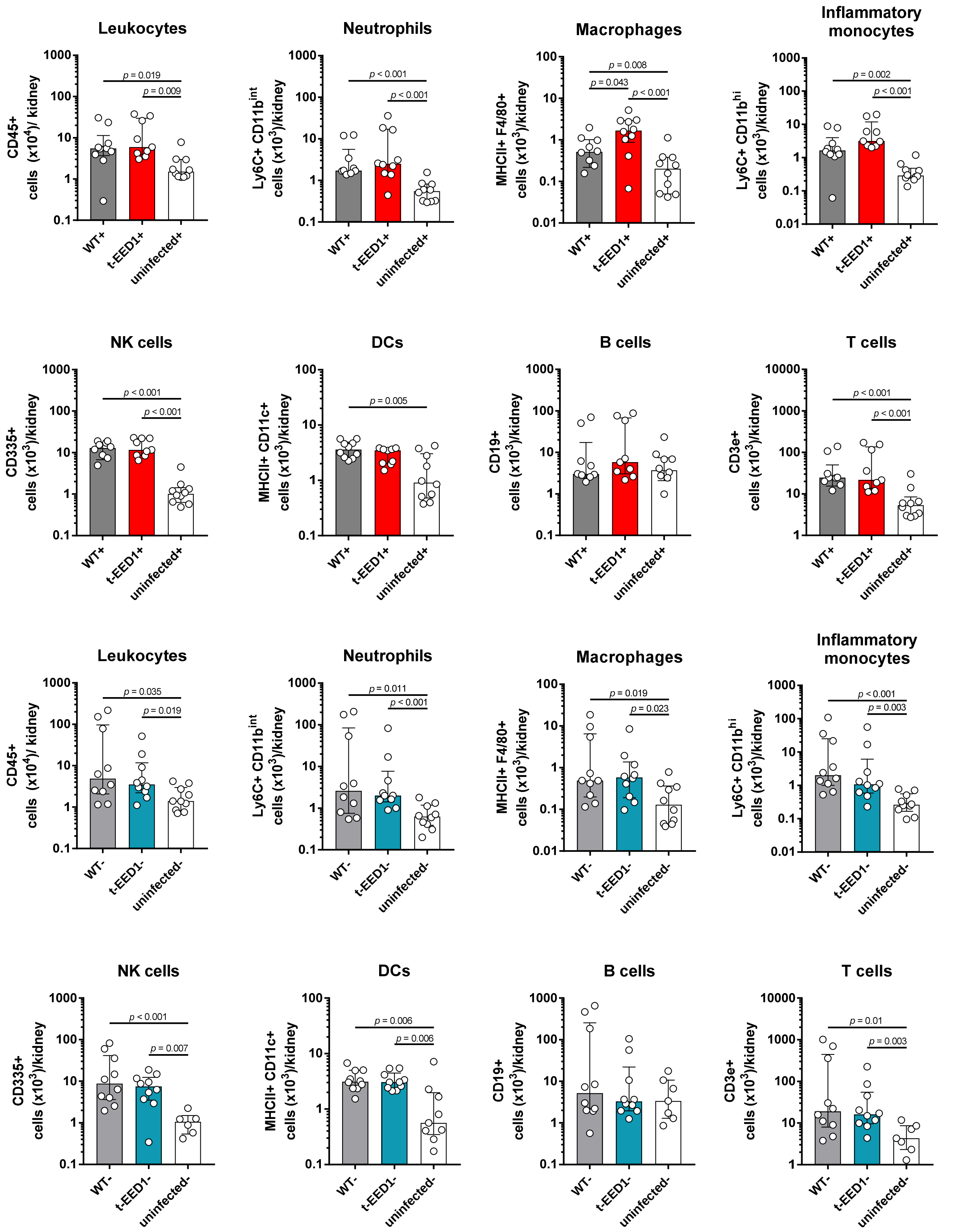

Supplementary Fig. 7 Renal leukocyte infiltration in mice intravenously infected with $2.5x10^4$ CFU/g body weight of WT or t-EED1 and in uninfected controls in the presence (+) or absence of doxycycline (-) 48 h post infection. The absolute number of living immune cells per kidney is shown as median and interquartile range. Two independent experiments , n=10, except for t-EED1+ n=9 and uninfected- NK cells n=6, DCs n=9, B cells and T cells n=7. Two-sided Mann-Whitney test, *p*-values are shown in the graphs. Source data are provided as a Source Data file.

Kidney infiltrating leukocytes 72 h p.i.

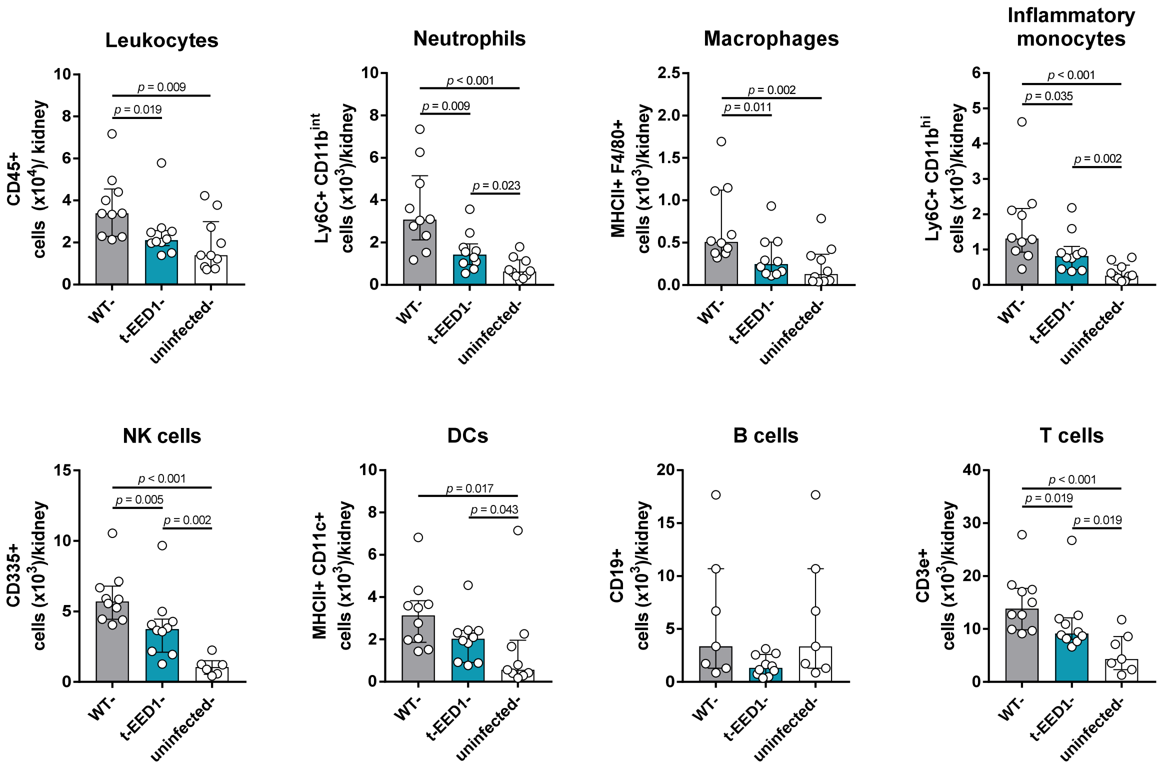

Supplementary Fig. 8 Renal leukocyte infiltration in mice intravenously infected with $2.5x10^4$ CFU/g body weight of WT or t-EED1 and in uninfected controls in absence of doxycycline (-) 72 h post infection. The absolute number of living immune cells per kidney is shown as median and interquartile range. Two independent experiments , n=10, except for uninfected- NK cells n=6, DCs n=9, B cells and T cells n=7. Two-sided Mann-Whitney test, *p*-values are shown in the graphs. Source data are provided as a Source Data file.

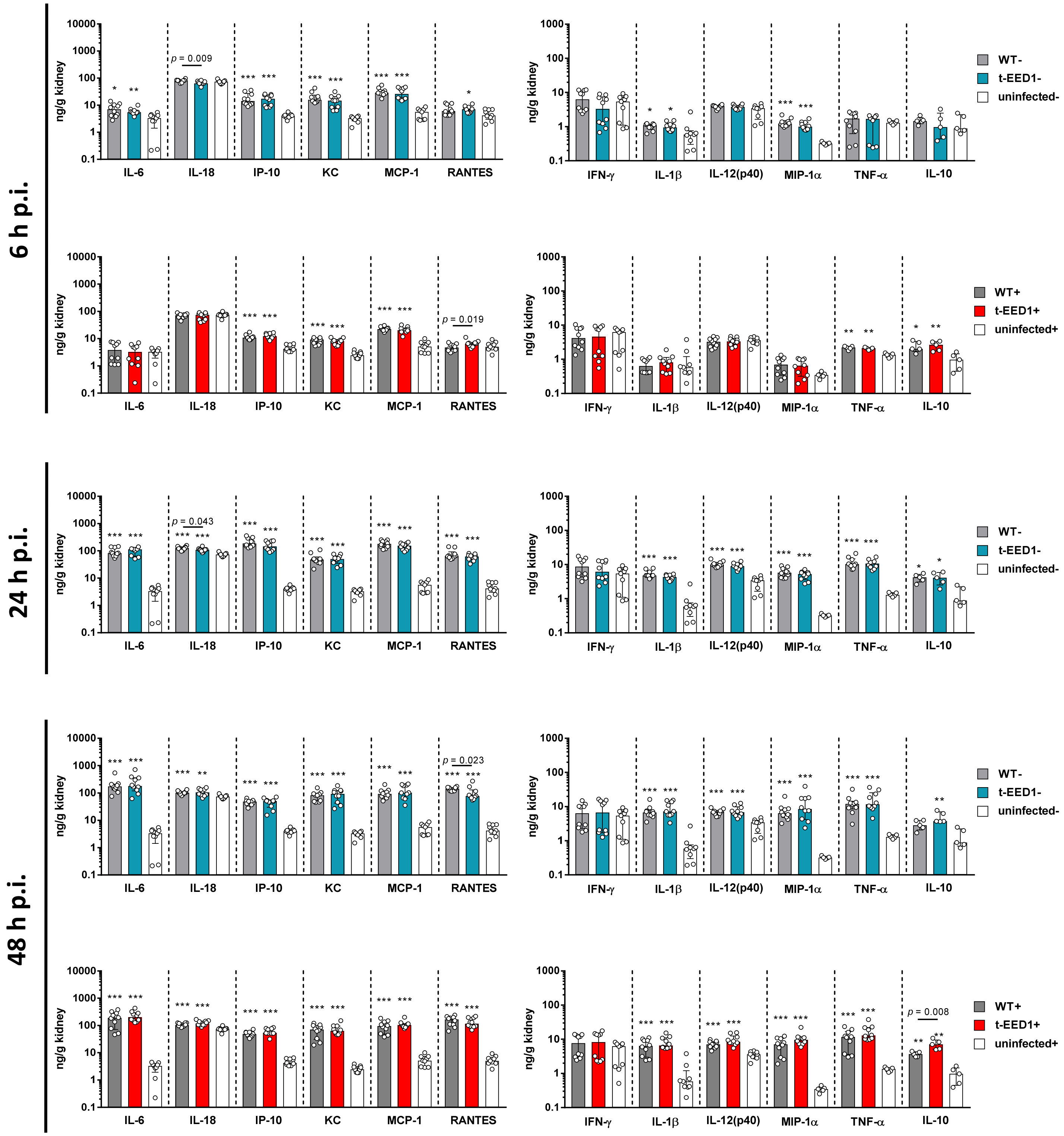

Supplementary Fig. 9 Local cytokine production in kidneys of mice intravenously infected with $2.5x10^4$ CFU/g body weight of WT or t-EED1 6, 24, 48 h post infection and in uninfected controls in the presence (+) or absence of doxycycline (-). Cytokine concentrations were determined in kidney homogenates. Shown is the median with interquartile range from two independent experiments. n=10, except for uninfected controls n=9; MIP-1α, TNF-α uninfected controls n=5. IL-10 data derived from one experiment, n=5. Two-sided Mann-Whitney test. Asterisks above bars represent significant differences compared to the uninfected control, * $p \leq 0.05$; ** $p \leq 0.01$; *** $p \leq 0.001$. Source data are provided as a Source Data file.

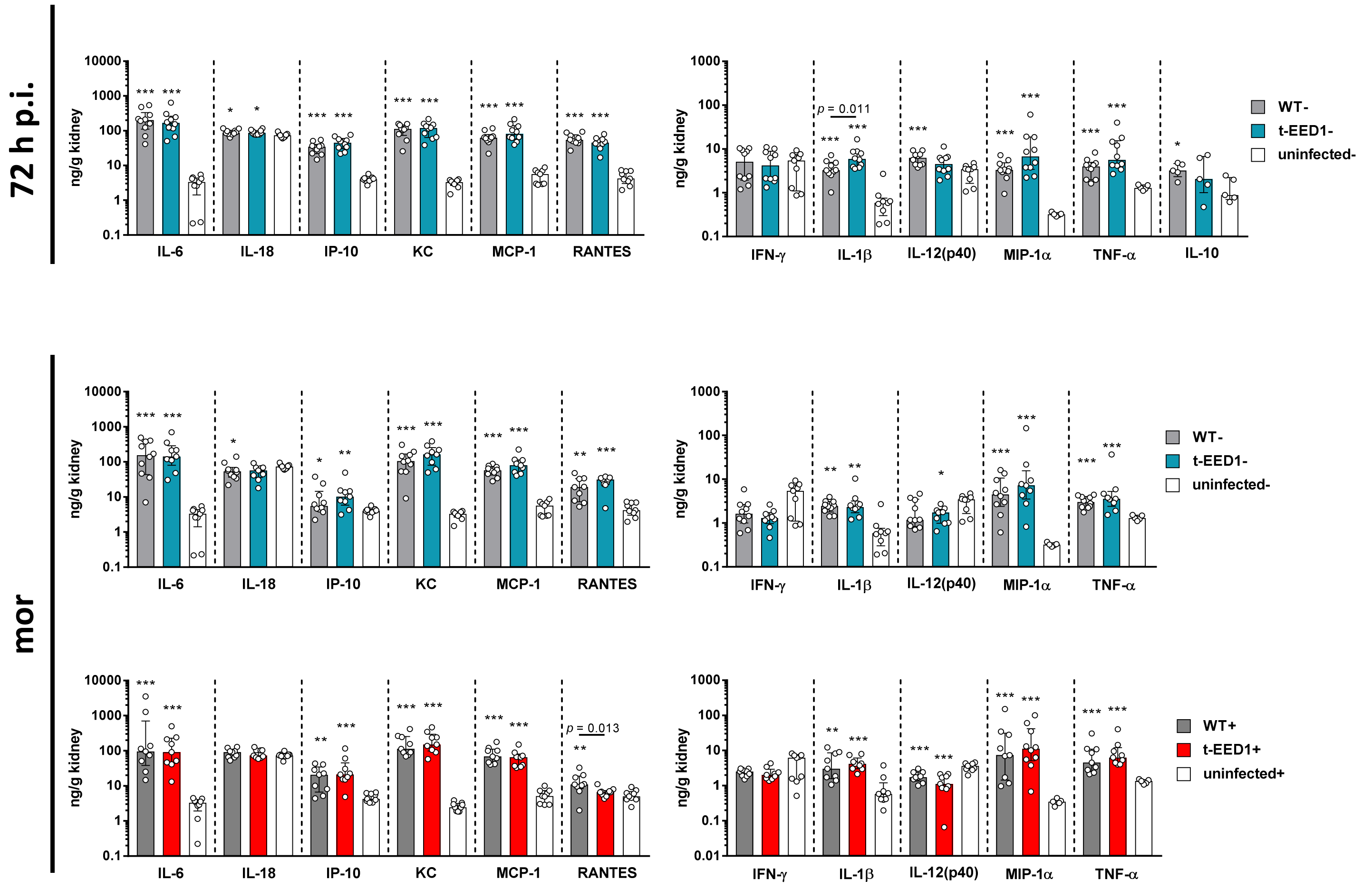

Supplementary Fig. 10 Local cytokine production in kidneys of mice intravenously infected with 2.5x10^4 CFU/g body weight of WT or t-EED1 72 h post infection, in moribund animals (mor) and in uninfected controls in the presence (+) or absence of doxycycline (-). Cytokine concentrations were determined in kidney homogenates. Shown is the median and interquartile range from two independent experiments. n=10, except for uninfected controls n=9; MIP-1α, TNF-α uninfected controls n=5. IL-10 data derived from one experiment, n=5. Two-sided Mann-Whitney test. Asterisks above bars represent significant differences compared to the uninfected control, * $p \le 0.05$; ** $p \le 0.01$; *** $p \le 0.001$. Source data are provided as a Source Data file.

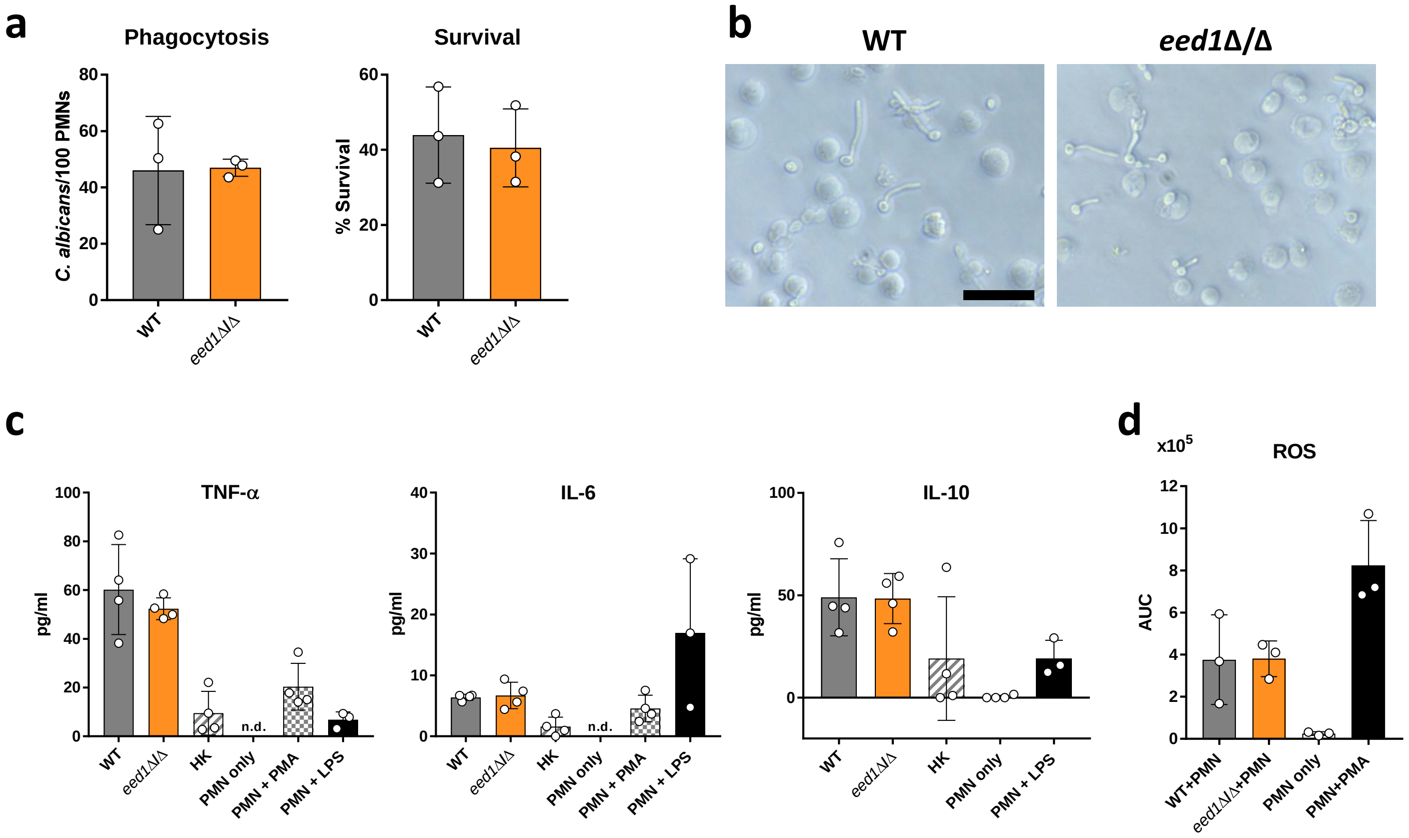

Supplementary Fig. 11 Interaction of *C. albicans* WT and *eed1*Δ/Δ mutant with murine bone marrow neutrophils (PMNs). PMNs were infected at a MOI of 1. a) The phagocytic index is the number of *C. albicans* cells phagocytosed by 100 PMNs within 1 h of incubation at 37 °C and 5% CO_2. Fungal survival was analyzed after co-incubation with PMNs at 37 °C and 5% CO_2 for 2 h by CFU plating. Survival was normalized to *C. albicans* controls incubated in the absence of immune cells. Graphs show the mean ±SD of three independent biological replicates. b) Morphology of *C. albicans* strains during co-incubation with PMNs after 3 h. Representative pictures of two biologically independent experiments are shown. Scale bar represents 20 µm and applies to all images. c) Cytokines released by PMNs after co-incubation for 24 h with living or heat-killed (HK) *C. albicans* WT cells. PMNs were left untreated (PMN only) as negative or stimulated with 100 nM PMA or 100 ng/ml LPS as positive controls. d) Release of reactive oxygen species (ROS) by PMNs upon stimulation with *C. albicans* was quantified by luminol-enhanced chemiluminescence assay in 2.5 min intervals over a period of 190 min using a microplate reader. The graph shows the calculated area under the curve (AUC). As negative and positive control, PMNs were left untreated or stimulated with 100 nM PMA, respectively. Shown is the mean ±SD from a,d) three or c) four independent biological replicates except for PMN+LPS n=3. Source data are provided as a Source Data file.

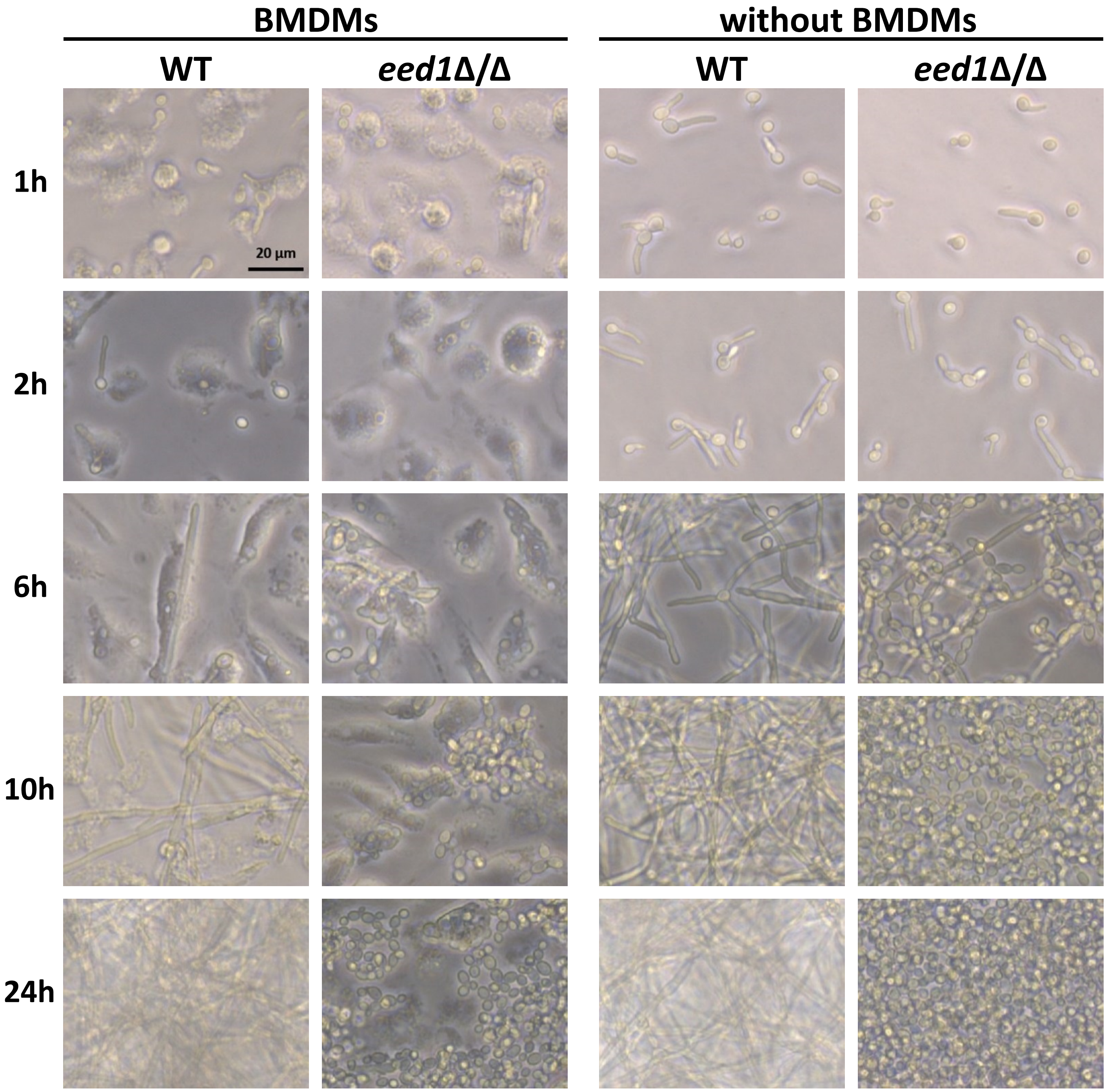

Supplementary Fig. 12 Morphology of *C. albicans* WT and *eed1*Δ/Δ mutant in the presence of bone marrow-derived macrophages (BMDMs; left) or without BMDMs (right). BMDMs were infected at a MOI of 1 and cells were incubated at 37 °C and 5% CO_2. Representative pictures of three biologically independent experiments are shown. Pictures were taken after various time points as indicated on the left. Scale bar applies to all images.

Supplementary Fig. 13

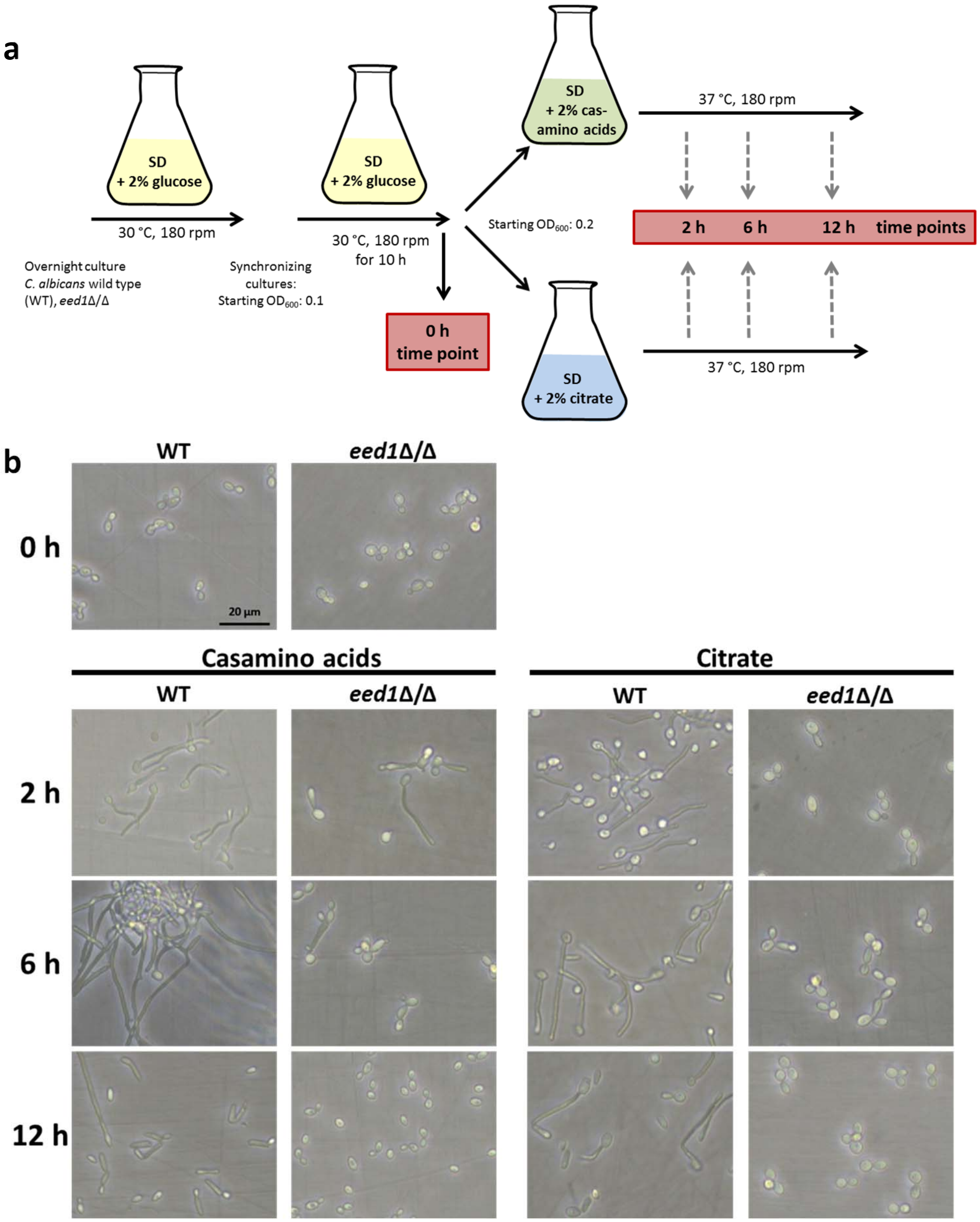

Supplementary Fig. 13 Sample preparation for RNAseq analysis and fungal morphology at the time of sampling. a) *C. albicans* WT (SC5314) and *eed1*Δ/Δ mutant were grown over night in Synthetic Minimal Media (SD) containing glucose as sole carbon source at 30 °C and 180 rpm. Cultures were synchronized in the same media and cultivated for 10 h at 30 °C (0 h). Cultures were then shifted to SD medium containing either casamino acids (CAA) or citrate as sole carbon source and incubated at 37 °C and 180 rpm. After 2 h, 6 h and 12 h samples were taken. b) Morphology of *C. albicans* WT (SC5314) and *eed1*Δ/Δ mutant during growth on citrate or casamino acids as sole carbon source at 37 °C and 180 rpm. Representative pictures of three biologically independent experiments are shown. The scale bar applies to all images.

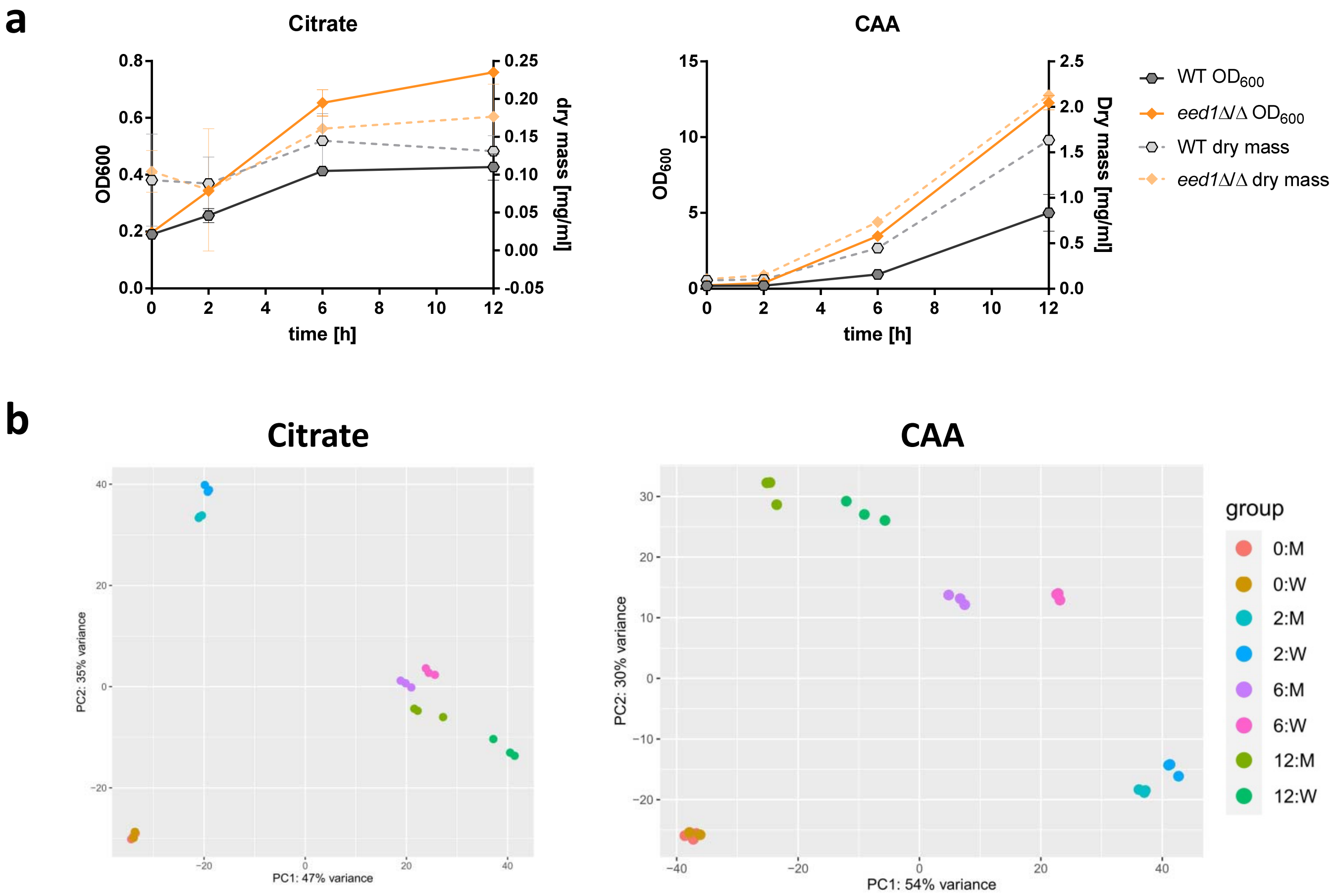

Supplementary Fig. 14 The *eed1*Δ/Δ mutant grows better on the alternative carbon sources citrate and casamino acids and shows differences in expression profiles compared to the WT. a) Growth of WT (SC5314) and *eed1*Δ/Δ mutant on citrate and casamino acids as sole carbon source at 37 °C and 180 rpm was recorded by OD measurement at 600 nm and the determination of dry mass. Mean ± SD from three biologically independent experiments (one flask per strain and experiment) are shown. Source data are provided as a Source Data file. b) Principal component analysis (PCA) of expression data obtained by RNAseq from *C. albicans* WT (W; SC5314) and *eed1*Δ/Δ mutant (M) grown on citrate or casamino acids for 0, 2, 6 and 12 h.

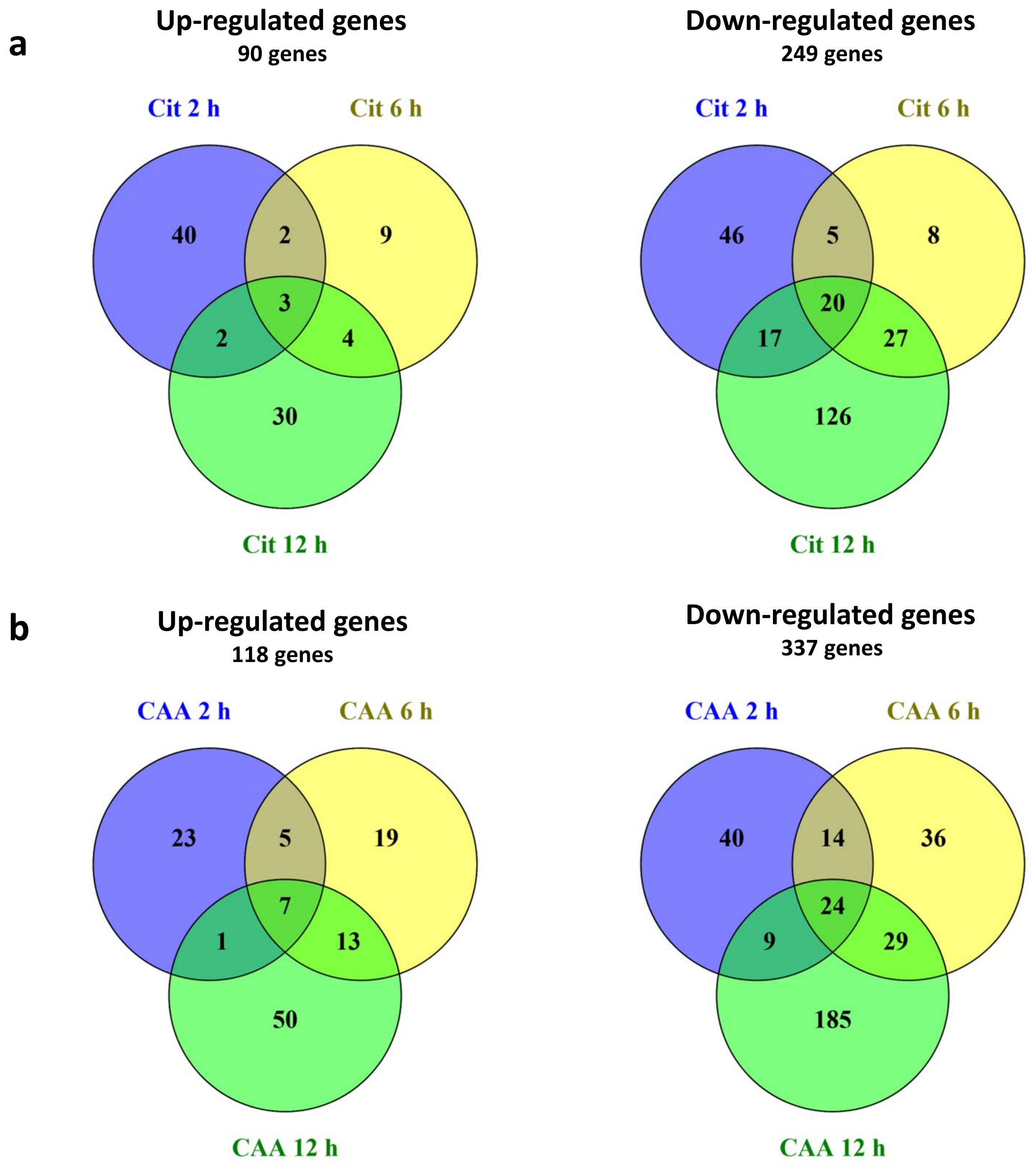

Supplementary Fig. 15 Venn diagrams showing the number of genes significantly up- and down-regulated (+/- $\log_2$ 2 and adjusted *p*-value < 0.05) in the *eed1*Δ/Δ mutant compared to WT (SC5314) during growth for 2, 6 and 12 h with a) citrate (Cit) or b) casamino acids (CAA) as sole carbon source at 37 °C.

Supplementary Fig. 16

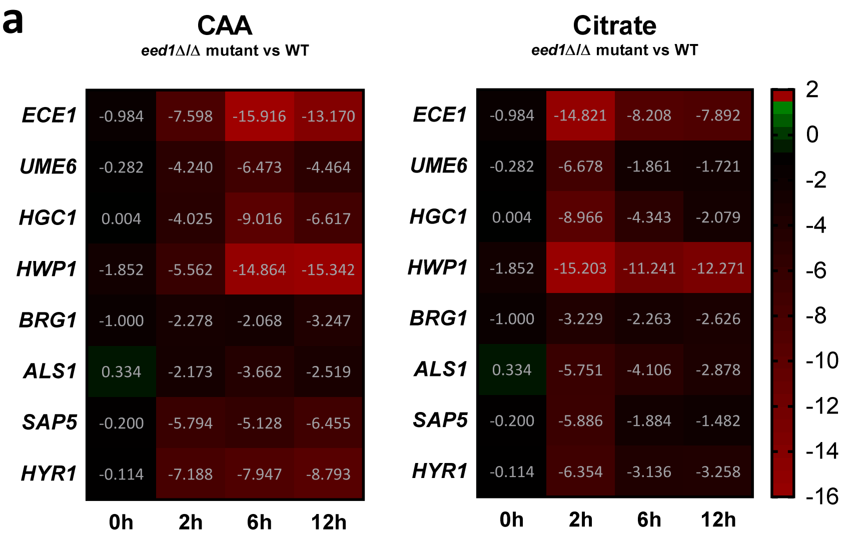

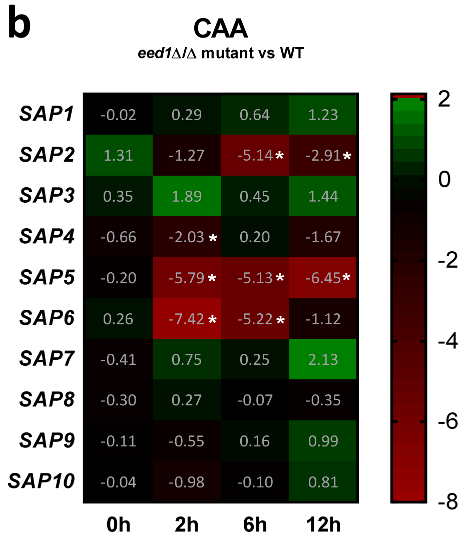

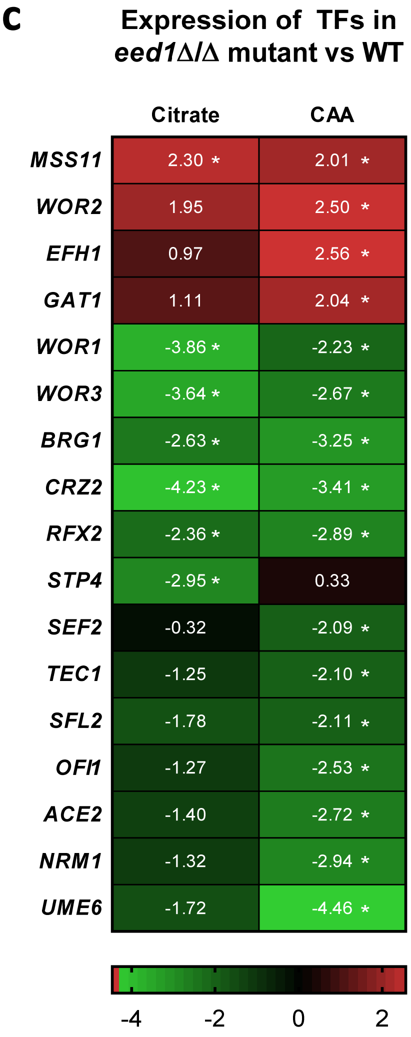

Description
activator that binds to Flo8 via a LisH motif to cooperatively activate transcription of hypha-specific genes; required for hyphal growth
regulator of white-opaque switching; required for maintenance of opaque state; Hap43-induced
minor role in transcriptional regulation vs Efg1; regulates filamentous growth, phenotypic switch; EFG1 and EFH1 genetically interact; expression interferes with mouse intestinal tract colonization
regulator of nitrogen utilization; required for nitrogen catabolite repression and utilization of isoleucine, tyrosine and tryptophan N sources; required for virulence in a mouse systemic infection model
Transcription factor ("master switch") of white-opaque phenotypic switching; required to establish and maintain the opaque state
Transcription factor; modulator of white-opaque switch; induced in opaque cells; promoter bound by Wor1
recruits Hda1 to hypha-specific promoters
important for gut growth
Transcriptional repressor; regulator of filamentation
colony morphology-related gene regulation by Ssn6
required for normal resistance to copper; repressed by Sfu1 in high-iron conditions; regulated by Sef1, Sfu1, and Hap43
white cell pheromone response, hyphal gene regulation; required for Spider and RPMI biofilm formation; regulates BCR1
required for filamentous growth, for virulence in RHE model but not in mice
Putative transcription factor with zinc finger DNA-binding motif, involved in regulation of white-opaque switching and filamentous growth
regulates morphogenesis, cell separation, adherence, virulence in a mice; mutant is hyperfilamentous
Transcriptional regulator of cell cycle gene expression
regulates translational efficiency and controls transition to filamentous growth

Supplementary Fig. 16 Heat plots showing transcriptional regulation of a) hypha-associated genes in *eed1*Δ/Δ mutant compared to WT (SC5314) in medium containing casamino acids (CAA; left) or citrate (right) as sole carbon source after different time points; b) the secreted aspartic proteinase (*SAP*) gene family in *eed1*Δ/Δ mutant compared to WT (SC5314) during growth with casamino acids as sole carbon source; c) transcription factors (TFs) in *eed1*Δ/Δ mutant compared to WT (SC5314) after 12 h of growth with citrate or casamino acids (CAA). The description is modified from the "Candida Genome Database" (http://www.candidagenome.org/). b,c) Asterisks indicate significant regulation (±$\log_2$ 2 and adjusted *p*-value < 0.05) as determined by two-sided Wald test, *p*-values were corrected for multiple comparisons using the Benjamini and Hochberg method.

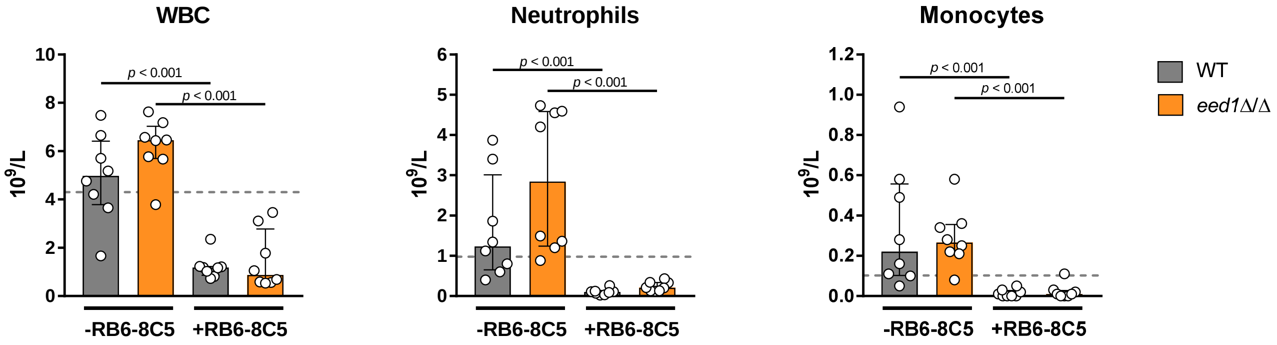

Supplementary Fig. 17 Neutrophils and monocytes were successfully depleted in RB6-8C5 treated mice. Immunocompetent or RB6-8C5 treated mice were systemically infected with 1x10^3 CFU/g body weight of WT (SC5314) or *eed1*Δ/Δ mutant. Absolute numbers of white blood cells (WBC), neutrophils and monocytes determined at the humane endpoint are shown. One experiment, n=8 mice per group. Dashed lines indicate median values from 10 uninfected immunocompetent mice. Shown is the median and interquartile range, two-sided Mann-Whitney test. *p*-values are shown in the graphs. Source data are provided as a Source Data file.

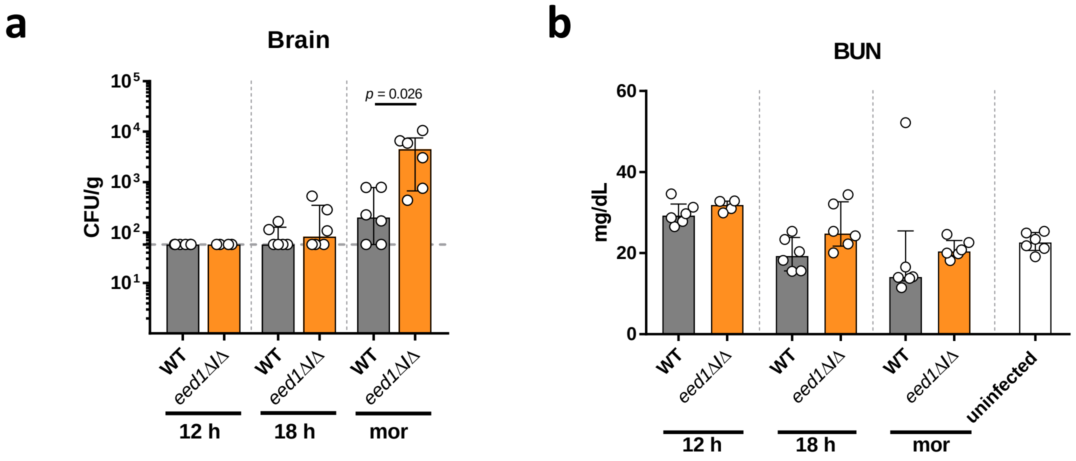

Supplementary Fig. 18 Fungal burden in brain and determination of kidney function in immunosuppressed mice after systemic infection. Mice depleted of neutrophils and monocytes by using the antibody RB6-8C5 were infected with $1x10^2$ CFU/g body weight of *C. albicans* WT (SC5314) or *eed1*Δ/Δ mutant. Mice were humanely sacrificed 12 and 18 h post infection and a) organ fungal burden was determined by CFU plating. Kidney function was measured by quantification of blood urea nitrogen (BUN) in serum of mice. Uninfected mice served as control (ctr). a,b) Two independent experiments, n=6, except for *eed1*Δ/Δ mutant at 12 h, n=4. Shown is the median with interquartile range, two-sided Mann-Whitney test. *p*-values are shown in the graphs. Source data are provided as a Source Data file.

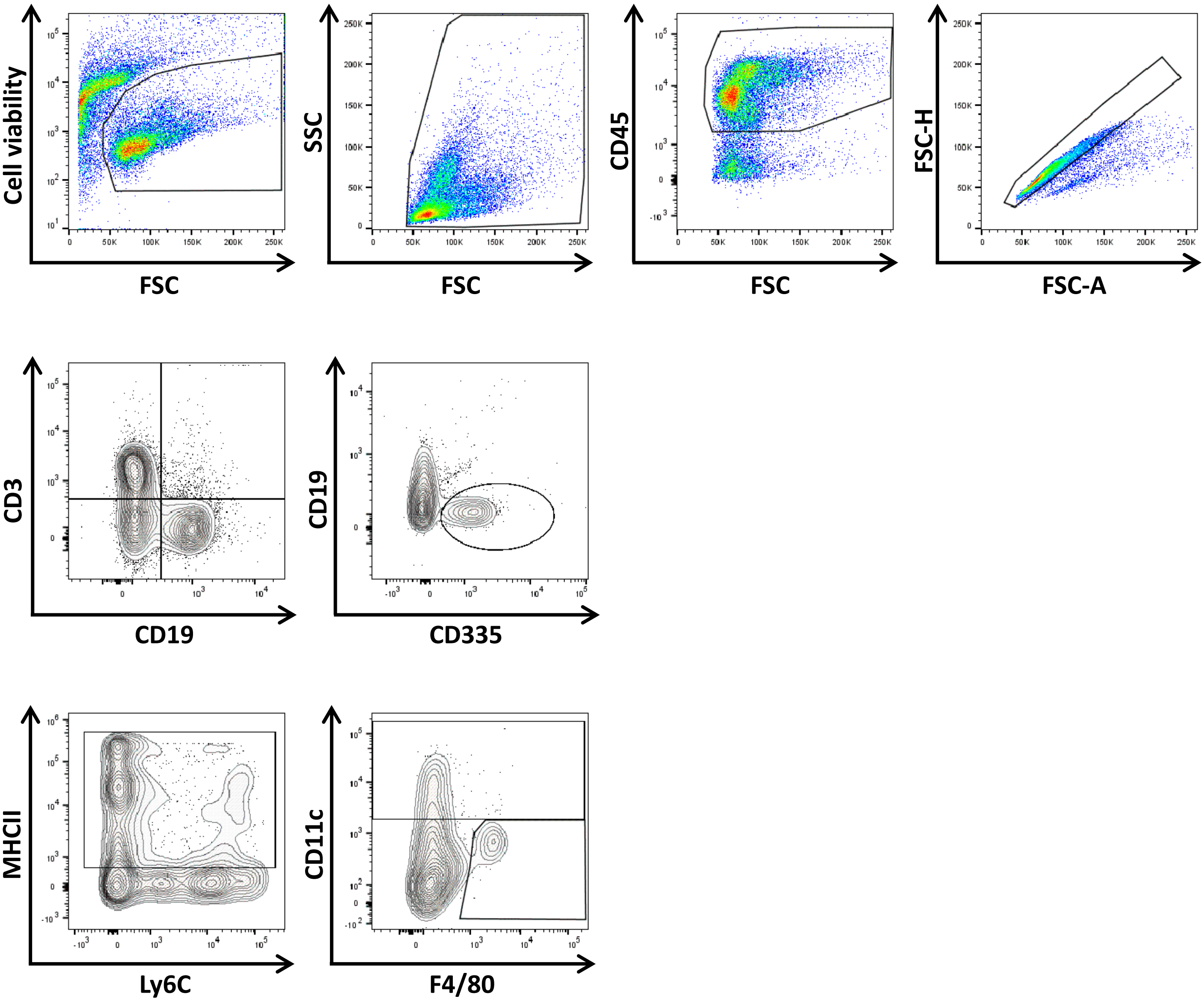

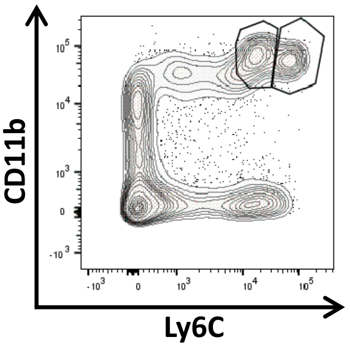

Leukocytes: living, CD45+, single cells
T cells: living, CD45+, single, CD3+ cells
B cells: living, CD45+, single, CD19+ cells
Dendritic cells (DCs): living, CD45+, single, MHCII+, CD11c+ cells
Macrophages: living, CD45+, single, MHCII+, CD11c-, F4/80+ cells
Neutrophils: living, CD45+, single, CD11b+, $LyC6^{intermediate}$
Inflammatory monocytes: living, CD45+, single, CD11b+, $LyC6^{high}$
Natural killer cells (NKs): living, CD45+, single, CD335+

Supplementary Fig. 19 Gating strategy for the differentiation of immune cell populations after flow cytometry. Dead cells were excluded from analysis by staining with the Fixable Viability Dye eFluor® 506. Living Fixable Viability Dye^{low} cells were gated on size and granularity to exclude debris. CD45+ single cells were used to further discriminate different immune cell populations. T and B cells were identified by expression of CD3 and CD19, respectively. Dendritic cells were identified by expression of MHCII and CD11c and macrophages were characterized as MHCII+, CD11c-, F4/80+ cells. Neutrophils and inflammatory monocytes were identified by co-expression of CD11b and Ly6C and differentiated by the level of Ly6C expression: neutrophils as $Ly6C^{int}$ and inflammatory monocytes as $Ly6C^{hi}$ cells. Natural killer cells (NKs) were identified by expression of CD335.

Supplementary Table 1: Primers used in this study

Name	Primer sequences (5’ → 3’)	Used for
**Eed1-TET-F**	GCTTTAACTTTCACTTTCATTTTCAATTTTGTTTGCTGCTTGTCTAACAGTTTATTATCATTTTGCTTTTTTTTTCATACTACAATTAACTACAA**GTAATACGACTCACTATAGGG**	Amplification of *URA3*- TR promoter region from p99CAU1 (Nakayama et al 2000)
**Eed1-TET-R**	ggatctgggagtcttacgaggacggcctgttccgtttctaatgttggatgtgttaaattgtcttctttccatgttgaaatccaaacgagaatgatgaag**CTAGTTTTCTGAGATAAAGCT**	Amplification of *URA3*- TR promoter region from p99CAU1 (Nakayama et al 2000)
