# Supplementary material for: Rapid proliferation due to better metabolic adaptation results in full virulence of a filament-deficient Candida albicans strain
Source: Nat Commun. 2021 Jun 23;12:3899. doi: 10.1038/s41467-021-24095-8 (PMC8222383; doi:10.1038/s41467-021-24095-8)
Supplement: Supplementary file 3 — Description of Additional Supplementary Files [file 41467_2021_24095_MOESM3_ESM.pdf]

Description of Additional Supplementary Files

File Name: Supplementary Data 1
Description: Genes significantly up- or down-regulated (+/- log_2 2 and adjusted p-value <0.05) in the *eed1*Δ/Δ mutant compared to WT (SC5314) with citrate or CAA as sole carbon source after 12 h of growth.
