# Supplementary material for: Rapid proliferation due to better metabolic adaptation results in full virulence of a filament-deficient Candida albicans strain
Source: Nat Commun. 2021 Jun 23;12:3899. doi: 10.1038/s41467-021-24095-8 (PMC8222383; doi:10.1038/s41467-021-24095-8)
Supplement: Supplementary file 5 — Reporting Summary [file 41467_2021_24095_MOESM5_ESM.pdf]

nature research

Corresponding author(s): Ilse D. Jacobsen

Last updated by author(s): May 12, 2021

Reporting Summary

Nature Research wishes to improve the reproducibility of the work that we publish. This form provides structure for consistency and transparency in reporting. For further information on Nature Research policies, see our Editorial Policies and the Editorial Policy Checklist.

Statistics

For all statistical analyses, confirm that the following items are present in the figure legend, table legend, main text, or Methods section.

n/a	Confirmed	
☐	☒	The exact sample size (*n*) for each experimental group/condition, given as a discrete number and unit of measurement
☐	☒	A statement on whether measurements were taken from distinct samples or whether the same sample was measured repeatedly
☐	☒	The statistical test(s) used AND whether they are one- or two-sided<br>*Only common tests should be described solely by name; describe more complex techniques in the Methods section.*
☒	☐	A description of all covariates tested
☐	☒	A description of any assumptions or corrections, such as tests of normality and adjustment for multiple comparisons
☐	☒	A full description of the statistical parameters including central tendency (e.g. means) or other basic estimates (e.g. regression coefficient) AND variation (e.g. standard deviation) or associated estimates of uncertainty (e.g. confidence intervals)
☐	☒	For null hypothesis testing, the test statistic (e.g. *F*, *t*, *r*) with confidence intervals, effect sizes, degrees of freedom and *P* value noted<br>*Give P values as exact values whenever suitable.*
☒	☐	For Bayesian analysis, information on the choice of priors and Markov chain Monte Carlo settings
☐	☒	For hierarchical and complex designs, identification of the appropriate level for tests and full reporting of outcomes
☒	☐	Estimates of effect sizes (e.g. Cohen's *d*, Pearson's *r*), indicating how they were calculated

Our web collection on statistics for biologists contains articles on many of the points above.

Software and code

Policy information about availability of computer code

Data collection

The following standard software provided by instrument suppliers was used for data collection:
Flow cytometry: BD FACSuite v.1.0.6
Multiplex cytokine measurements: Luminex MAGPIX System, Luminex xPONENT software v4.2
Plate reader (OD, cytokine ELISAs, LDH): Tecan infinite MPlex reader, i-control v2.0 software
Microscopy: ZEN lite 2011 software
Immunhistochemistry: PROGRES GRYPHAX® software version 1.1.8.153
Hematology: Mindray BC-5300Vet Auto Hematology Analyzer, Application Software V01.07.00.15500

Data analysis	GraphPad Prism 7 was used to analyze all data sets, except flow cytometry and RNAseq data.<br>Flow cytometry data was analyzed using FlowJo V.10.0.8 software.<br><br>For analysis of RNAseq data, the following software tools were used:<br>Mapping of the fastq files and counting of the gene transcriptions was performed using the European Galaxy server (https://usegalaxy.org) that provides an environment and sets of tools for analyses.<br>The following tools on the Galaxy server were used: FastQC, Cutadapt, RNA-Star, FeatureCount. These are functions included in the Deseq2 package; Deseq2 package version 1.26.0 was used.<br>Analyses in R were performed in R version 3.6.0<br>Gene Ontology (GO) term enrichment analysis was performed using the CGD GO Term Finder (http://www.candidagenome.org/help/goTermFinder.shtml); this software/database does not provide version numbers; it was accessed in January 2021. The same applies to REVIGO (http://revigo.irb.hr/). For the creation of graphs and diagrams, cytoscape version 3.7.2 (https://cytoscape.org/) and Venny2.1 (https://bioinfogp.cnb.csic.es/tools/venny/) were used.

For manuscripts utilizing custom algorithms or software that are central to the research but not yet described in published literature, software must be made available to editors and reviewers. We strongly encourage code deposition in a community repository (e.g. GitHub). See the Nature Research guidelines for submitting code & software for further information.

Data

Policy information about availability of data

All manuscripts must include a data availability statement. This statement should provide the following information, where applicable:

- Accession codes, unique identifiers, or web links for publicly available datasets
- A list of figures that have associated raw data
- A description of any restrictions on data availability

The RNAseq data that support the findings of this study are available at NCBI under BioProject accession number PRJNA714826. Source data are provided with this paper.

Field-specific reporting

Please select the one below that is the best fit for your research. If you are not sure, read the appropriate sections before making your selection.

[x] Life sciences [ ] Behavioural & social sciences [ ] Ecological, evolutionary & environmental sciences

For a reference copy of the document with all sections, see nature.com/documents/nr-reporting-summary-flat.pdf

Life sciences study design

All studies must disclose on these points even when the disclosure is negative.

Sample size	Statistical power of sample sizes was calculated using GraphPad StatMate based on previous observations regarding data variability.
Data exclusions	no data was excluded; in one experiment (Fig. 3) one mouse assigned to the WT group had to be removed before the start of the experiment due to malocclusion of the incisors leading to reduced food intake. In another experiment (Fig. 4) one control mouse was removed before the start of the experiment due to unspecific symptoms.
Replication	All experiments were repeated at least once (unless indicated otherwise in the manuscript and explained below) on separate occasions. Where significant differences between groups were observed in the pooled data, differences also occurred in all individual experiments, indicating that differences were reproducible.<br><br>Some animal experiments were performed only once for one of the following reasons:<br>1) Systemic (i.v.) infections: A set of experiments with a different infection dose each was performed. Each experiment with a distinct dose was performed independently of the others (at a different time, with a different batch of mice, and independently grown infection inoculum). The experiments with different doses were compared to assess if (i) differences between groups were dose-dependent and (ii) if differences between groups were consistent across different doses. As results (different survival between group A and group B, differences in fungal burden) were consistent across the different doses used, they were considered reproducible. Thus, and in line with the 3R concept, these sets of experiments were not repeated.<br><br>2) Systemic (i.v.) and intraperitoneal infections: Instead of repeating an experiment with the same fungal strains (wildtype and mutant), a second independent experiment was performed using fungal mutants deficient for the same gene but constructed independently with a different method; consistent results obtained with independent mutants on separate occasions were considered as indication that the result was successfully replicated.<br><br>3) The colonization experiment with immunocompetent mice was performed only once, but with two cohorts (cages) per treatment group: One cohort was sacrificed on day 4, the other on day 14. Fecal fungal load in both cohorts of the same treatment group was comparable for the first two time points and thus considered reproducible. For colonization with the wildtype and cyclophosphamide treatment, the data obtained in the single experiment is comparable to other experiments conducted earlier in a different context - thus, this type of experiment was only repeated for the mutant to determine if the higher kidney burden would be reproducible.

Randomization	Upon arrival, animals were randomly picked from the transport box and distributed between all cages within each individual experiment. Mean and SD of body weight were calculated per cage on day 1 after arrival to determine if this parameter was similar between cages.<br><br>For other types of experiments (cell culture infection or infection experiments with primary cells), all cells to be used per experiment were pooled and the pool was used for inoculation of microtiter plates. The strains were not randomly assigned to wells, but preliminary experiments (using one infection strain only) were performed to test for technical bias associated with location of wells on plates. As a result, the outer wells of 96-well plates were generally excluded from use in all cell culture experiments.<br><br>No technical issues requiring randomization on multiwell plates were observed for other types of analyses.
Blinding	Formal blinding of animal experiments was not feasible because the local authorities mandate that all experimental details are indicated on the cage cards and made available to all personnel involved in animal care and/or experimental procedures. We aimed to nonetheless achieve some level of blinding by performing clinical monitoring in random cage order and documentation by cage number (rather than strain designation). Likewise, sample designation (for subsequent analyses such as histology or flow cytometry) was based on numerical experiment designation and cage/animal numbers without inclusion of specific group designations (strain) on the sample tubes or slides.

Reporting for specific materials, systems and methods

We require information from authors about some types of materials, experimental systems and methods used in many studies. Here, indicate whether each material, system or method listed is relevant to your study. If you are not sure if a list item applies to your research, read the appropriate section before selecting a response.

Materials & experimental systems

n/a	Involved in the study	
	x	Antibodies
	x	Eukaryotic cell lines
x		Palaeontology and archaeology
	x	Animals and other organisms
x		Human research participants
x		Clinical data
x		Dual use research of concern

Methods

n/a	Involved in the study	
x		ChIP-seq
	x	Flow cytometry
x		MRI-based neuroimaging

Antibodies

Antibodies used	The following antibodies were used (antibody, supplier, clone name, # catalogue no., lot no., concentration/dilution, unique identifiers provided by Resource Identification Portal (RRIDs))<br><br>In vivo cell depletion: InVivoPlus anti-mouse Ly6G/Ly6C (Gr-1) monoclonal antibody, BioXcell, RB6-8C5, # BP0075, lot 682618O1, 100 µg/ mouse, RRID AB_10312146<br><br>Flow cytometry:<br>PerCP anti-mouse CD45, BD Biosciences, 30-F11, # 557235, lot 8179968, 0.2 µg/10^6 cells, RRID AB_396609<br>APC anti-mouse CD11b, eBioscience, M1/70, # 17-0112, lot E07074-1636, 0.06 µg/10^6 cells, RRID AB_469343<br>eFluor 450 anti-mouse CD335, eBioscience, 29A1.4, # 48-3351, lot 1952458, 0.25 µg/10^6 cells, RRID AB_10557245<br>FITC anti-F4/80, eBioscience, BM8, # 11-4801, lot2007710, 0.25 µg/10^6 cells, RRID AB_263719<br>PE anti-CD11c, eBioscience, N418, # 12-0114, lot 2146031, 0.25 µg/10^6 cells, RRID AB_465552<br>PE-Cy7 anti-MHCII, eBioscience, M5/114.15.2, # 25-5321, lot 2018404, 0.1 µg/10^6 cells, RRID AB_10870792<br>eFluor 450 anti-Ly-6C, eBioscience, HK1.4, # 48-5932, lot 4277890, 0.25 µg/10^6 cells, RRID AB_10805519<br>FITC anti-CD19, BD Biosciences, 1D3, # 553785, lot 5015949, 0.125 µg/10^6 cells, RRID:AB_395049<br>PE-Cy7 anti-mouse CD3e, eBioscience, 145-2C11, # 25-0031, lot 1993626, 0.2 µg/10^6 cells, RRID AB_469572<br>Blocking antibody: anti-mouse CD16/32, BioLegend, 93, # 101320, several lots used (a Certificate of Analysis is provided by the manufacturer for each lot), 1:50, RRID:AB_1574975<br><br>Immunhistochemistry:<br>anti-Digoxigenin as part of the ApopTag in situ apoptosis detection kit, EMD Millipore, affinity purified sheep polyclonal antibody, # S7100, lot 3163956, concentration as specified in the detection kit instructions
Validation	InVivoPlus anti-mouse Ly6G/Ly6C (Gr-1) monoclonal antibody (clone RB6-8C5; BioXcell), manufacturer information: The RB6-8C5 monoclonal antibody reacts strongly with mouse Ly6G and weakly with mouse Ly6C previously referred to as GR-1. Ly6G is a 21-25 kDa member of the Ly-6 superfamily of GPI-anchored cell surface proteins with roles in cell signaling and cell adhesion. Ly6G is expressed differentially during development by cells in the myeloid lineage including monocytes macrophages granulocytes and neutrophils. Monocytes typically express Ly6G transiently during development while mature granulocytes and peripheral neutrophils retain expression making Ly6G a good cell surface marker for these populations.The RB6-8C5 antibody has been shown to inhibit the binding of the 1A8 antibody. The 1A8 monoclonal antibody reacts specifically with mouse Ly6G with no reported cross reactivity with

Ly6C.
This study: Cell depletion in vivo was confirmed by hematology.

PerCP anti-CD45 (30-F11, BD Biosciences), manufacturer information: The 30-F11 clone has been reported to react with all isoforms and both alloantigens of CD45, which is found on hematopoietic stem cells and all cells of hematopoietic origin, except erythrocytes. CD45 is a transmembrane glycoprotein which is expressed at high levels on the cell surface, and its presence distinguishes leukocytes from non-hematopoietic cells. Applications tested by the manufacturer: flow cytometric analysis of mouse bone marrow cells and splenocytes.

FITC anti-CD19 (1D3, BD Biosciences), manufacturer information: The 1D3 antibody reacts with CD19, a B lymphocyte-lineage differentiation antigen. CD19 is expressed throughout B-lymphocyte development from the pro-B cell through the mature B-cell stages. Terminally differentiated plasma cells do not express CD19. CD19 has also been detected on peritoneal mast cells, co-localized with CD21/CD35, and it is proposed to play a role in complement-mediated mast-cell activation. Applications Reported: flow cytometric analysis. Applications tested by the manufacturer: flow cytometric analysis of mouse bone marrow cells and splenocytes.

APC anti-CD11b (M1/70, eBioscience): manufacturer information: The M1/70 monoclonal antibody reacts with mouse CD11b, the 165-170 kDa integrin alphaM. CD11b non-covalently associates with CD18 to form alphaMbeta2 integrin (Mac-1) and binds to CD54 (ICAM-1), C3bi, and fibrinogen. Mac-1 is expressed by macrophages, NK cells, granulocytes, activated lymphocytes and mouse B-1 cells in the peritoneal cavity. M1/70 is also cross-reactive to human CD11b, and can be used for the detection of this antigen on human peripheral blood monocytes, granulocytes, and a subset of NK cells. Applications tested: The M1/70 antibody has been tested by flow cytometric analysis of mouse splenocytes. This can be used at less than or equal to 0.125 µg per test. A test is defined as the amount (µg) of antibody that will stain a cell sample in a final volume of 100 µL. Cell number should be determined empirically but can range from 10^5 to 10^8 cells/test.

eFluor anti-CD335 (29A1.4, eBioscience), manufacturer information: The monoclonal antibody 29A1.4 recognizes mouse NKp46, also known as CD335. Expression of CD335 is uniquely found on NK cells (including immature NK cells, defined as DX5- CD3-, and thereby allowing discrimination between NKT cells and NK cells (NKp46+, CD3-). Furthermore, unlike many of the NK markers which also stain NKT cells, staining with 29A1.4 is not strain specific. Staining has been shown on C57Bl/6, SJL, CBA/CA and BALB/C strains. Applications Tested: This 29A1.4 antibody has been tested by flow cytometric analysis of mouse splenocytes. This can be used at less than or equal to 0.5 µg per test. A test is defined as the amount (µg) of antibody that will stain a cell sample in a final volume of 100 µL. Cell number should be determined empirically but can range from 10^5 to 10^8 cells/test.

FITC anti-F4/80 (BM8, eBioscience), manufacturer information: The BM8 monoclonal antibody reacts with mouse F4/80 antigen, an approximately 160 kDa surface receptor. The F4/80 antigen is expressed by a majority of mature macrophages and is one of the best markers for this population of cells. However, other cell types, such as peritoneal eosinophils, Langerhans cells, and some other dendritic cell subtypes, have been reported to express this antigen as well. Expression of F4/80 commences during early myeloid development in vivo and can be upregulated on BM cells stimulated in vitro with M-CSF. Some populations of macrophages, especially in the lymphoid microenvironment, may be devoid of F4/80. Applications Tested: The BM8 antibody has been tested by flow cytometric analysis of mouse resident peritoneal exudate cells. This can be used at less than or equal to 0.5 µg per test. A test is defined as the amount (µg) of antibody that will stain a cell sample in a final volume of 100 µL. Cell number should be determined empirically but can range from 10^5 to 10^8 cells/test.

PE anti-CD11c (N418, eBioscience), manufacturer information: The N418 monoclonal antibody reacts with mouse CD11c, the integrin alphaX. CD11c non-covalently associates with beta2 integrin to form the CD11c/CD18 heterodimer. CD11c is expressed by dendritic cells, a subset of Intestinal Intraepithelial Lymphocytes (IEL) and some activated T cells. N418 binds to CD11c on splenic dendritic cells in the T-dependent areas of mouse spleen. Applications tested: The N418 antibody has been tested by flow cytometric analysis of mouse splenocytes. This can be used at less than or equal to 0.5 µg per test. A test is defined as the amount (µg) of antibody that will stain a cell sample in a final volume of 100 µL. Cell number should be determined empirically but can range from 10^5 to 10^8 cells/test.

PE-Cy7 anti-MHCII (M5/114.15.2 eBioscience), manufacturer information: The M5/114.15.2 monoclonal antibody reacts with the mouse major histocompatibility complex class II, both I-A and I-E subregion-encoded glycoproteins (I-A b, I-A d, I-A q, I-E d, I-E k, not I-A f, I-A k, or I-A s). It detects a polymorphic determinant present on B cells, monocytes, macrophages, dendritic cells, and activated T lymphocytes from mice carrying the H-2 b, H-2 d, H-2 q, H-2 p, H-2 r and H-2 u but not from mice carrying the H-2 s or H-2 f haplotypes. The M5/114 mAb is reported to inhibit I-A-restricted T cell responses of the H-2 b, H-2 d, H-2 q, H-2 u but not H-2 f, H-2 k, or H-2 s haplotypes. Applications tested: This M5/114.15.2 antibody has been tested by flow cytometric analysis of mouse splenocytes. This can be used at less than or equal to 0.25 µg per test. A test is defined as the amount (µg) of antibody that will stain a cell sample in a final volume of 100 µL. Cell number should be determined empirically but can range from 10^5 to 10^8 cells/test.

eFluor anti-Ly-6C (HK1.4, eBioscience), manufacturer information: The monoclonal antibody HK1.4 recognizes mouse Ly-6C, a GPI-linked protein of the Ly6 family. Ly-6C is found on monocytes/macrophages, endothelial cells and granulocytes as well as a subset of lymphocytes. Some variation of expression is found on different mouse strains in regards to expression on CD4 and CD8 lymphocytes. These correlate to 2 alleles both of which are recognized by HK1.4: Ly6c.1 found on C57Bl/6 and SJL cells which results in staining of both CD4 and CD8 cells while Ly6-C.2 found on BALB/c and 3H/He results in staining of CD8, but not CD4 cells. In vitro addition with HK1.4 antibody can increase proliferation and stimulate cytokine release. Applications tested: This HK1.4 antibody has been tested by flow cytometric analysis of mouse splenocytes. This can be used at less than or equal to 0.25 µg per test. A test is defined as the amount (µg) of antibody that will stain a cell sample in a final volume of 100 µL. Cell number should be determined empirically but can range from 10^5 to 10^8 cells/test.

PE-Cy7 anti-CD3e (145-2C11, eBioscience), manufacturer information: The 145-2C11 monoclonal antibody reacts with mouse CD3e, a 20 kDa subunit of the TCR complex. CD3 is expressed by thymocytes in a developmentally regulated manner and by all mature T cells. 145-2C11 is commonly used as a phenotypic marker for mouse T cells. Applications tested: This 145-2C11 antibody has been tested by flow cytometric analysis of mouse thymocytes and splenocytes. This can be used at less than or equal to 1 µg per test. A test is defined as the amount (µg) of antibody that will stain a cell sample in a final volume of 100 µL. Cell number should be determined

empirically but can range from 10^5 to 10^8 cells/test.

anti-mouse CD16/32 (93; BioLegend) - used as blocking antibody; manufacturer information: CD16 is the low affinity IgG Fc receptor III (FcR III) and CD32 is FcR II. CD16/CD32 are expressed on B cells, monocytes/macrophages, NK cells, granulocytes, mast cells, and dendritic cells. The Fc receptors bind antibody-antigen immune complexes and mediate adaptive immune responses. TruStain FcX™ is specific to the common epitope of CD16/CD32. It is useful for blocking non-specific binding of immunoglobulin to the Fc receptors. Each lot of this antibody is quality control tested by immunofluorescent staining with flow cytometric analysis. Reactivity: mouse. Recommended Usage: For blocking of Fc receptors in flow cytometric analysis, pre-incubate the cells with TruStain FcX™ at 1.0 µg per 10^6 cells in 100 µl volume for 5-10 minutes on ice prior to immunostaining. It is not necessary to wash cells between these blocking and immunostaining steps.

Fixable Viability Dye eFluor® 506 (eBioscience), manufacturer information: Fixable Viability Dye eFluor™ 506 is a viability dye that can be used to irreversibly label dead cells prior to cryopreservation, fixation and/or permeabilization procedures. Unlike 7-AAD and propidium iodide, cells labeled with Fixable Viability Dyes can be washed, fixed, permabilized, and stained for intracellular antigens without any loss of staining intensity of the dead cells. Thus, using Fixable Viability Dyes allows dead cells to be excluded from analysis when intracellular targets are being studied. Fixable Viability Dyes may be used to label cells from all species. Applications tested: pre-titrated and tested by flow cytometric analysis of mouse thymocytes.

anti-Dioxigenin: supplied as part of the ApopTag in situ apoptosis detection kit (EMD Millipore). The ApopTag Peroxidase In Situ Apoptosis Detection Kit detects apoptotic cells in situ by by the TUNEL method. Used in several studies to quantify apoptosis in murine tissues, examples: doi: 10.1016/j.jcmgh.2019.10.008. , doi: 10.1038/s41418-020-0532-1 , doi: 10.1038/s41413-019-0073-8

Testing of antibody panels with appropriate isotype controls was done during method establishment. Fluorescence compensation was performed before each experiment.

Eukaryotic cell lines

Policy information about cell lines

Cell line source(s)	HepaRG: Gibco; A498: DSMZ; TR146: Episkin; Caco-2 clone type C2BBe1: ATCC (ATCC®CRL-2102)
Authentication	Cell lines were ordered from the sources indicated above and kept as stocks in liquid nitrogen after 2-4 rounds of propagation. Expression of epithelial and mesenchymal markers was analysed by fluorescence microscopy in random batches. Gross morphological analysis was performed before each experiment.
Mycoplasma contamination	All cell lines were tested regularly (at least 2x/year) and were found to be free of Mycoplasma contamination.
Commonly misidentified lines (See ICLAC register)	No commonly misidentified cell lines were used in the study.

Animals and other organisms

Policy information about studies involving animals; ARRIVE guidelines recommended for reporting animal research

Laboratory animals	The following information is stated in the manuscript: Eight- to ten-week-old female specific-pathogen-free BALB/c mice (16 to 18 g), purchased from Charles River (Germany), were housed in groups of five in individually ventilated cages at 22 ± 1°C, 55 ± 10 % relative humidity, 12h/12h dark/light cycle, with free access to food and water and autoclavable mouse houses as environmental enrichment.
Wild animals	No wild animals were used in the study.
Field-collected samples	No field collected samples were used in the study.
Ethics oversight	Protocols and animal experiments were approved by the Thuringian authority and ethics committee (Thüringer Landesamt für Verbraucherschutz, permit numbers: HKI-19-003, 03-007/13, 03-002/11, 03-004/15, 03-008/13).

Note that full information on the approval of the study protocol must also be provided in the manuscript.

Flow Cytometry

Plots

Confirm that:

- [x] The axis labels state the marker and fluorochrome used (e.g. CD4-FITC).
- [x] The axis scales are clearly visible. Include numbers along axes only for bottom left plot of group (a 'group' is an analysis of identical markers).
- [x] All plots are contour plots with outliers or pseudocolor plots.
- [x] A numerical value for number of cells or percentage (with statistics) is provided.

Methodology

Sample preparation	Analysis of immune cell abundance in mouse organs: Organs were cut into small pieces and digested in the presence of collagenase D (30 µg/ml; Sigma Aldrich) and DNase I (0.7 mg/ml; Sigma Aldrich) in RPMI (RPMI 1640; Gibco) supplemented with 10% fetal bovine serum (FBS; Bio&SELL), Pen Strep (100 U/ml Penicillin and 100 µg/ml Streptomycin; Life Technologies), and 1 mM sodium pyruvate (Gibco) for 30 min at 37 °C with moderate horizontal shaking (70 rpm). Single cells were obtained by passing the digested tissue through a 70 µm cell strainer. Cells were washed and erythrocytes were lysed by addition of red blood cell lysis buffer (0.15 mM NH4Cl, 10 mM KHCO3, 1 mM Na2EDTA, pH 7.2). Remaining cells were washed, resuspended in 70% Percoll (GE Healthcare) and layered under 30% Percoll. Leukocytes were enriched by density gradient centrifugation (400 x g, 20 min, room temperature (RT), acceleration 1, deceleration 0). Leukocytes were collected from the interphase, washed with PBS and volumes were determined. Cells were transferred in a 96-well plate and stained.
Instrument	FACSVerse (BD Biosciences)
Software	FlowJo V.10.0.8
Cell population abundance	no sorting was performed; purity of neutrophils isolated by Percoll gradient was analysed by flow cytometry (details provided in the manuscript)
Gating strategy	Dead cells were excluded from analysis by staining with the Fixable Viability Dye eFluor® 506. Living Fixable Viability Dye low cells were gated on size and granularity to exclude debris. CD45+ single cells were used to further discriminate different immune cell populations. T and B cells were identified by expression of CD3 and CD19, respectively. Dendritic cells were identified by expression of MHCII and CD11c and macrophages were characterized as MHCII+, CD11c-, F4/80+ cells. Neutrophils and inflammatory monocytes were identified by co-expression of CD11b and Ly6C and differentiated by the level of Ly6C expression: neutrophils as Ly6C int and inflammatory monocytes as Ly6C hi cells. Natural killer cells (NKs) were identified by expressing CD335.

☒ Tick this box to confirm that a figure exemplifying the gating strategy is provided in the Supplementary Information.
